# A spatial theory for emergent multiple predator–prey interactions in food webs

**DOI:** 10.1002/ece3.3250

**Published:** 2017-07-28

**Authors:** Tobin D. Northfield, Brandon T. Barton, Oswald J. Schmitz

**Affiliations:** ^1^ Centre for Tropical Environmental and Sustainability Studies College of Marine and Environmental Sciences James Cook University Cairns QLD Australia; ^2^ Department of Biological Sciences Mississippi State University Starkville MS USA; ^3^ School of Forestry and Environmental Studies Yale University New Haven CT USA

**Keywords:** Predation, competition, habitat domain, biodiversity, niche

## Abstract

Predator–prey interaction is inherently spatial because animals move through landscapes to search for and consume food resources and to avoid being consumed by other species. The spatial nature of species interactions necessitates integrating spatial processes into food web theory and evaluating how predators combine to impact their prey. Here, we present a spatial modeling approach that examines emergent multiple predator effects on prey within landscapes. The modeling is inspired by the habitat domain concept derived from empirical synthesis of spatial movement and interactions studies. Because these principles are motivated by synthesis of short‐term experiments, it remains uncertain whether spatial contingency principles hold in dynamical systems. We address this uncertainty by formulating dynamical systems models, guided by core habitat domain principles, to examine long‐term multiple predator–prey spatial dynamics. To describe habitat domains, we use classical niche concepts describing resource utilization distributions, and assume species interactions emerge from the degree of overlap between species. The analytical results generally align with those from empirical synthesis and present a theoretical framework capable of demonstrating multiple predator effects that does not depend on the small spatial or temporal scales typical of mesocosm experiments, and help bridge between empirical experiments and long‐term dynamics in natural systems.

## 1 INTRODUCTION

There is growing recognition that developing a predictive understanding of predator–prey interactions in food webs cannot be fully understood without deliberately considering the spatial domain over which interactions take place (Amarasekare, 2007; Barraquand & Murrell, 2013; Holt, 2002; McCann, Rasmussen, & Umbanhowar, 2005; Schmitz, 2007). Interactions are inherently spatial because fundamentally animals move through landscapes to search for and consume food resources and to avoid being consumed by other species (Amarasekare, 2007; McCann et al., 2005). But, the way predator–prey interactions play out can be complex, depending on the modular nature of the food web (e.g., intraguild predation, exploitative competition, apparent competition, keystone predation), the habitat structure of landscapes, and the relative mobility of the interacting species (Amarasekare, 2007; Barraquand & Murrell, 2013; McCann et al., 2005; Schmitz, 2007).

Theoretical efforts to examine spatial food web dynamics have followed two main modeling approaches. The first—patch modeling—begins by imposing habitat patch structure onto landscapes. It then examines how food web dynamics emerge from species moving between habitat patches according to assumptions about species’ mobility, and predator–prey interactions within habitat patches determined by the structure of the food web module in which the species are configured (Amarasekare, 2007; McCann et al., 2005). The second—spatially explicit movement modeling—begins with contiguous landscapes. It then examines the emergence of patchy spatial structure and food web dynamics that result from individuals of predator and prey species moving and interacting locally according to specified rules of engagement (Barraquand & Murrell, 2013).

The patch modeling approach has thus far been most appealing because it offers straightforward analytical tractability that can lead to generalizable principles. These principles in turn can motivate empirical tests in a variety of study systems. But, the underlying patchwork landscape assumed by the theory may not apply universally. In many cases, species movement and interaction can be generalized as occurring over contiguous landscapes; or if habitat patchiness exists, the spatial grain of species movement and interaction is sufficiently coarse that spatial heterogeneity can be reasonably abstracted. The alternative, spatially explicit movement modeling may conform better to analyses on more contiguous landscapes. But being a complex simulation approach, prediction relies on rules of engagement or empirical parameter estimates idiosyncratic to specific study systems. As such, it does not yet lend itself to making general predictions for different study systems (Barraquand & Murrell, 2013). Hence the desire for more general analytical formalisms that characterize the emergence of spatial heterogeneity due to species’ spatial movement and interaction in contiguous landscapes.

Here, we present a spatial modeling approach that examines emergent multiple predator effects on prey within contiguous landscapes. The modeling is inspired by the habitat domain concept that was derived from empirical synthesis of studies of spatial movement and interactions by a variety of invertebrate and vertebrate predator and prey species (Northfield, Crowder, Jabbour, & Snyder, 2012; Schmitz, 2005, 2007, 2010). Habitat domain describes the spatial extent of habitat space that predators and prey use in the course of their resource selection within what can be approximated as a contiguous landscape space. As such, it is useful for understanding how the spatial juxtaposition of predators and prey can lead to emergent multiple predator effects and how these effects are altered by environmental change (e.g., Barton & Schmitz, 2009; Schmitz & Barton, 2014).

## 2 MODELING FRAMEWORK

### 2.1 The concept of habitat domain, spatial food web modules, and multiple predator effects

**Figure 1:**
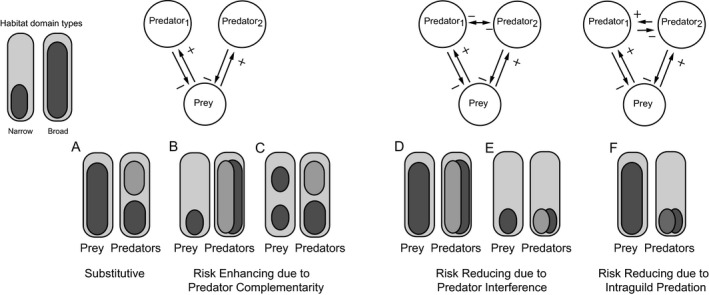
Considerations of ways multiple predator species may affect a common prey species through spatial interactions determined by predator and prey habitat domains. Rectangles represent habitat space available, and ovals represent habitat occupancy. Species can have either narrow or broad habitat domains. In the absence of interference behavior and intraguild predation, predators are expected to have substitutable effects when both predators and prey have broad habitat domains (a). In contrast, risk‐enhancing (complementary) effects are expected when predators have spatially juxtaposed, narrow domains if their prey have broad domains, but prey rarely move between habitats (b). Risk‐enhancing effects can also occur whenever the prey domain is narrow and predator domains are broad (c). Predators are expected to have risk‐reducing effects due to interference when prey and predator habitat domains are all similar (d, e), and due to intraguild predation whenever prey have a broad domain and predators have narrow, overlapping habitat domains and different hunting modes (f)

Habitat domain is a way to conceptualize how predators and prey should interact as a consequence of contingencies in their spatial movement and overlap. An individual's habitat domain can be narrow or broad (Fig. 1). Predator hunting mode (e.g., ambush, sit‐and‐pursue, or active) determines the spatial extent of predator movement (i.e., habitat domain). Within a given environmental context, habitat domain size is consistent among predators with similar hunting modes (Miller, Ament, & Schmitz, 2014). The distribution of the predator population then also depends on the habitat suitability along an environmental gradient, which can lead to a broad distribution of predators even if they rarely move along the gradient. Therefore, predator distribution breadth arises from reduced environmental specialization, and movement within the habitat domain, due to a more active hunting mode. Prey habitat domain size may depend on prey traits like diet breadth (specialist vs. generalist) and foraging mode (leaf chewing, grazing, and sap feeding) (Northfield, Snyder, Snyder, & Eigenbrode, 2012; Schmitz, 2010; Singer et al., 2014; Straub, Finke, & Snyder, 2008). Habitat domain size and spatial location in habitat space may also be malleable, as the biotic and abiotic environmental context changes (Barton & Schmitz, 2009; Schmitz & Barton, 2014).

Habitat domain can help to translate nonspatial food web modules into a spatial context to predict how different kinds of predator–prey interactions emerge (Northfield, Crowder et al., 2012; Schmitz, 2007). It underscores the importance of spatial context because it shows that there is not a 1:1 mapping between a specific food web module and the nature of the multiple predator effects on prey that emerge from interactions implied by the module. For example, multiple predators and prey could be configured into three classical types of nonspatial food web modules (Fig. 1): exploitative competition; interference competition; and intraguild predation. But there may be several different spatial juxtapositions of predators and prey for any given food web module, each of which may lead to different emergent multiple predator–prey interactions.

In a spatial context, exploitative competition could arise in three ways. In the first case, predator species are spatially segregated and vie for a shared prey species that moves between different spatial locations occupied by each predator (Fig. 1a). Here, the prey has a large habitat domain and the predators have small, adjacent domains. In the second case (Fig. 1b), the two predators converge on the habitat occupied by the shared prey, because the predators have large overlapping domains that overlap the prey with a small domain. In the third case (Fig. 1c), each predator species specializes on exploiting spatially separated populations of the shared prey. Empirical synthesis shows that these spatial configurations lead to different emergent multiple predator effects on prey. In the first case, predators have substitutive effects on prey because, by being in separate locations, one predator spatially compensates for the effects of the other predator (Schmitz, 2007). Hence, multiple predator effects on prey mortality should be compensatory and thereby the predators together neither enhance nor reduce the net mortality of prey. In the second case, predators have complementary effects. Both predators should increase mortality risk to prey, relative to their individual effects, leading to additive (or even multiplicative) mortality effects on the shared prey (Schmitz, 2007). In the third case, the two predators technically do not compete, but rather operate as separate food chains involving their spatially corresponding prey population (Northfield, Crowder et al., 2012). This scenario also enhances risk of mortality to the entire prey species across the landscape.

Predator interference arises when both predator species and prey occupy similar habitat domains. Spatially, this food web module may arise either when predators and prey all have large habitat domain (Fig. 1d), or when they all have small habitat domains (Fig. 1e) (Schmitz, 2007). In both cases, predators undergo interference competition because one predator species preempts the other from gaining access to the shared prey. Empirical synthesis shows that in this case, the predators reduce their net effects on the prey by engaging in interference interactions. Hence, risk of mortality to the prey becomes reduced by interspecific interactions between predators (Schmitz, 2007).

Intraguild predation arises when predator species have small, overlapping domains and prey have a large habitat domain (Fig. 1f). In this case, prey can use a spatial refuge to evade both predators (Schmitz, 2007). Consequently, without recourse to capture other prey, predators attack and consume each other. Hence, risk of mortality to prey is again reduced (Schmitz, 2007).

These principles of habitat domain and contingent multiple predator–prey interactions are motivated by synthesis of short‐term experiments in which predator and prey were permitted to move, but predator densities were generally held constant. Hence, it remains uncertain if the principles about spatial contingencies continue to hold in dynamical systems where there is interplay among changes in predator–prey interactions, movement, and abundance (Straub, Finke, & Snyder, 2008; Tylianakis & Romo 2010). We address this uncertainty by formulating dynamical systems models, guided by core habitat domain principles, to examine long‐term multiple predator–prey spatial dynamics.

### 2.2 Translating movement into habitat domain

**Figure 2:**
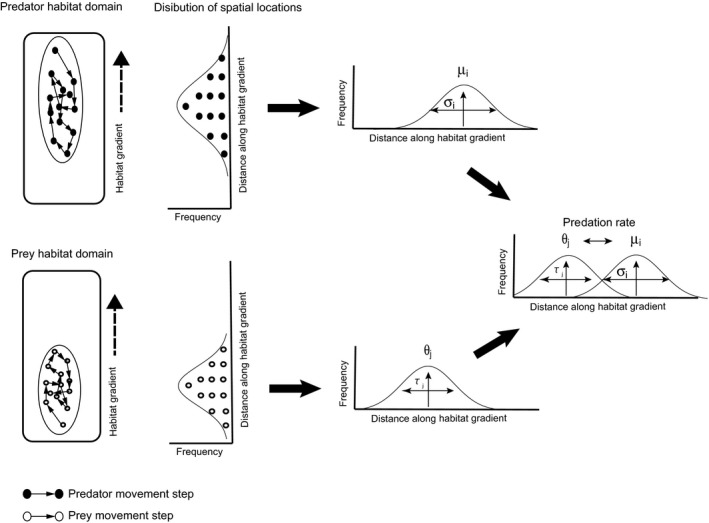
Translation from spatial movement behavior to calculation of species interactions. The utilization distribution is based on individual movement behavior within a habitat domain. Next, the movement within the habitat domain is translated into a frequency distribution of spatial occurrences—the utilization distribution—of each species. This utilization distribution forms the basis for modeling predator and prey population dynamics arising from spatial overlap and interactions. A similar approach is taken for describing intraguild interactions between predators

We characterize predator and prey spatial locations in contiguous space through the application of spatial utilization distributions (see fig. 1 in Barraquand & Murrell, 2013). Foraging and movement by an individual predator or prey through a contiguous habitat (or along a habitat gradient) can be depicted as sequential movement steps (Fig. 2). The spatial extent of all the foraging movement steps can be circumscribed using an ellipse (Fig. 2). That ellipse represents an individual's habitat domain (Fig. 2). Thus, habitat domain size is controlled by the variance of an individual predator's or prey's movement distribution across space. For example, individual sit‐and‐wait predators will have narrow habitat domains, by definition, and individual actively roaming hunting predators can have narrow or broad habitat domains.

The spatial locations of an individual within its habitat domain can then be translated into a frequency distribution of spatial occurrences—the utilization distribution (Barraquand & Murrell, 2013). An individual's utilization distribution is generated by plotting frequencies of spatial locations along a gradient within its habitat domain.

Deriving the population level utilization distribution is then merely a matter of aggregating the utilization distribution of each population member. The population habitat domain size can be estimated as the difference between the upper and lower limits of the population level utilization distribution. The population habitat domain could range from narrow to broad depending on where population members assort themselves spatially (e.g., locally clustered vs. broadly dispersed). There is certainly a wide range of within‐species behavioral ecological interactions that determine how population members become assorted across a habitat gradient (e.g., social groups vs. intraspecific territoriality) to determine population habitat domain size. However, for our purposes here, we will simply examine multiple predator–prey interactions based on utilization distribution breadths themselves, rather than explore how inherent within‐population behavioral ecology creates different habitat domain breadths.

The utilization distribution can be used as the basis for modeling predator and prey population dynamics arising from their spatial overlap and interactions. Thus, in our modeling, species interactions and dynamics emerge as a consequence of the degree of predator and prey spatial overlap, weighted by their frequency of spatial occurrence.

### 2.3 Model description

We adapt a mathematical formalism (see Abrams, 2000 for review) that has been used to describe one determinant of predator–prey interaction, namely trait‐matching of predation rates (e.g., Patel & Schreiber, 2015; Schreiber, Burger, & Bolnick, 2011). Such trait‐matching models express predator–prey interactions in terms of the frequency distribution of predator traits like body size or gape width that determine whether or not a predator can capture and consume a prey with a given size distribution. The idea of “trait‐matching” is adapted here by focusing on the frequency distributions of predator and prey space use—the utilization distributions (e.g., May, 2001). Hence, predation by a particular predator on a prey population depends on the spatial locations of the predator's habitat domain relative to that of the prey, and the respective domain sizes (as determined by the utilization distributions).

We assume that the utilization distribution of the predator and prey species follows a Gaussian distribution over the habitat gradient (Fig. 2). Predator population *i*'s utilization distribution is then centered spatially at μ_*i*,_ and prey population *j*'s utilization distribution is centered spatially at θ_*j*_ (Fig. 2). Because the upper and lower bounds of the entire fitted utilization distribution may not be finite for the prey and predators, we use the parameters τ_*j*_ and σ_*i*_, respectively, to describe the standard deviations of the frequency distributions, as surrogate measures of habitat domain size (Fig. 2). We assume that prey risk is uniform across the habitat (i.e., there are no undescribed environmental factors that covary with the habitat gradient such as relative prey cover). Thus, we assume that the attack rate for an individual predator of species *i* at location *x* on prey *j* is described by $a_{ij}=\alpha_{i}{\left(2\pi \tau_{j}^{2}\right)}^{-0.5}\text{exp}\left[-{\left(x-\theta_{j}\right)}^{2}/\left(2\tau_{j}^{2}\right)\right]$, where α_*i*_ describes the maximum predation rate for predator species *i*. This formalism permits analyses involving any number of predator and prey species. However, we examine dynamics within a two‐predator–prey structure. This is done deliberately in the interest of tractability to understand basic behavior of the dynamical systems comprised of species with different habitat domains.

Adapting the approach of Schreiber et al. (2011) and Patel and Schreiber (2015) and altering the models so that τ_*j*_ represents the prey standard deviation. The mean predation rate of predator species *i* on prey species *j*, ${\overset{¯}{a}}_{ij}$, is estimated by integrating over the probability density function, *p*(*x*_i_, μ_*i*_) describing the predator's utilization distribution across the habitat gradient:

$${\overset{¯}{a}}_{ij}=\int_{-\infty }^{\infty }a_{ij}\left(x\right)p\left(x,\mu \right)=\frac{\alpha_{i}}{\sqrt{2\pi (\sigma_{Pi}^{2}+\tau_{j}^{2})}}\text{exp}\left[\frac{-{\left(\mu_{i}-\theta_{j}\right)}^{2}}{2(\sigma_{Pi}^{2}+\tau_{j}^{2})}\right],$$

where *x* is the predator's spatial location along the habitat gradient. This function can be embedded into a general population dynamic formalism to describe rates of change in population sizes of multiple prey (*N*_*j*_) and predator (*P*_*i*_) species:

(1) $$\begin{array}{cc} \frac{dN_{j}}{dt} & =r_{j}N_{j}\left(1-\frac{N_{j}}{k_{j}}\right)-\sum_{i=1}^{2}{\overset{¯}{a}}_{ij}N_{j}P_{i}\\ \frac{dP_{i}}{dt} & =c_{i}\sum_{j=1}^{2}{\overset{¯}{a}}_{ij}N_{j}P_{i}-m_{i}P_{i}, \end{array}$$

where *k*_*j*_ and *r*_*j*_ are the carrying capacity and intrinsic growth rate for prey *j*, respectively, and *c*_*i*_ is rate of conversion of prey consumed to predators produced for predator species *i*, and *m*_*i*_ is basal mortality of predator *i*. This basic formalism assumes that there is no competition between prey species or prey populations. These equations then are a mathematical representation of the exploitative competition food web module (Fig. 1). They also lend themselves to be easily modified to represent interference competition and intraguild predation modules.

We generate a dynamical systems model to represent an interference food web module (Fig. 1) by assuming interference interactions increase predator species mortality from direct infliction of harm, or through increased energy expenditure that reduces the capacity to survive other mortality causes. In a two‐predator system, mortality rate due to interference from predator 1 on predator 2 is described as:

$$b_{i}=\frac{\beta }{\sqrt{2\pi (\sigma_{P1}^{2}+\sigma_{P2}^{2})}}\text{exp}\left[\frac{-{\left(\mu_{1}-\mu_{2}\right)}^{2})}{2(\sigma_{P1}^{2}+\sigma_{P2}^{2})}\right],$$

which follows traditional interference competition formalisms (e.g., May, 2001). We assume that interference increases mortality above baseline *m*_*i*_ such that total mortality is *m*_*i*_ + *b*_*i*_. Here, the parameter β describes the maximum rate of intraguild interactions between predators, which occurs when predator occupy the same location. The rates of change in the abundance of prey (*N*_*j*_), predator 1 (*P*_1_), and predator 2 (*P*_2_) are then described by:

(2) $$\begin{array}{cc} \frac{dN_{j}}{dt} & =rN_{j}\left(1-\frac{N_{j}}{k}\right)-\sum_{i=1}^{2}{\overset{¯}{a}}_{ij}N_{j}P_{i}\\ \frac{dP_{i}}{dt} & =c_{i}\sum_{j=1}^{2}a_{ij}N_{j}P_{i}-b_{i}P_{1}P_{2}-m_{i}P_{i}, \end{array}$$

To improve interpretability, we focus analyses on parameter values for which stable equilibriums exist. Detailed evaluations of similar models can be found elsewhere (e.g., Patel & Schreiber, 2015; Schreiber et al., 2011; Vanbaalen & Sabelis, 1993). Equilibrium solutions are presented in Appendix S1.

We generate an intraguild predation model by assuming one predator consumes the other, the intensity of which is determined by the degree of spatial overlap between the two predators. Thus, predation rate on predator 1 (intraguild prey) by predator 2 (intraguild predator), *b*_*p*_, is given by:

(3) $$\bar{b_{p}}=\int_{-\infty }^{\infty }b_{p}\left(x\right)p\left(x,\mu_{2}\right)=\frac{\beta }{\sqrt{2\pi (\sigma_{P1}^{2}+\sigma_{P2}^{2})}}\text{exp}\left[\frac{-{\left(\mu_{1}-\mu_{2}\right)}^{2}}{2(\sigma_{P1}^{2}+\sigma_{P2}^{2})}\right]$$

and hence the rates of change in the abundance of prey (*N*), predator 1 (*P*_1_), and predator 2 (*P*_2_) are described by

(4) $$\begin{array}{cc} \frac{dN_{j}}{dt} & =rN_{j}\left(1-\frac{N_{j}}{k}\right)-\sum_{i=1}^{2}a_{ij}N_{i}P_{j}\\ \frac{dP_{1}}{dt} & =c_{1}\sum_{j=1}^{2}a_{1j}N_{i}P_{1}-\bar{b_{p}}P_{1}P_{2}-m_{i}P_{1}\\ \frac{dP_{2}}{dt} & =c_{2}\sum_{j=1}^{2}a_{2j}N_{i}P_{2}+c_{b}\bar{b_{p}}P_{1}P_{2}-m_{i}P_{2}, \end{array}$$

where $\bar{b_{p}}$ is the intraguild predation rate, *c*_*b*_ is the conversion rate from intraguild prey consumed to intraguild predators produced, and all other parameters are as described in equation (1). To understand how multiple predator effects emerge from habitat use patterns, we evaluate the effects of the distance between predator and prey utilization distributions, and the breadth of prey habitat utilization distribution for the prey, τ. Particular scenarios were selected from conceptual models developed from empirical synthesis (Fig. 1; Northfield, Crowder et al., 2012; Schmitz, 2007).

We analyzed emergent multiple predator effects in our model by comparing prey densities in single versus multiple predator treatments for each habitat domain scenario once equilibrium states were reached. When a predator is present in a single‐species treatment, we assume the predator's resource utilization distribution is the same as in the corresponding multiple predator treatment. We measure the magnitude of emergent multiple predator effects as

(5) $$D_{T}=\frac{N_{\mathrm{Both}\_\mathrm{predators}}^{*}-\frac{1}{2}\sum_{k}N_{\mathrm{Predator}\_k}^{*}}{N_{\mathrm{Both}\_\mathrm{predators}}^{*}}$$

where N*_Both_predators_ is the prey density summed across both prey populations in the two‐predator equilibrium, and N*_Predator_k_ is the prey density in each of the two single‐predator equilibriums (Loreau, 1998). Negative *D*_*T*_ values indicate that there are fewer prey in more‐diverse predator communities than in single‐predator communities (i.e., prey risk enhancement), and positive *D*_*T*_ vales indicate that there are more prey (i.e., prey risk reduction). Values at or near zero are considered substitutable effects, where prey densities are similar for single and two‐predator equilibriums.

### 2.4 Model simulation

To understand the influence of changes in each resource utilization distribution in the model, we first found all of the equilibrium solutions analytically in MATLAB (MATLAB 8.5.0, The MathWorks Inc, Natick, MA, 2015). Next, we plotted the equilibrium solutions for changing values of the focal parameter. To evaluate the effects of dynamics where equilibrium solutions were unstable, we used the differential equation solver in the *R* package deSolve (Soetaert, Meysman, & Petzoldt, 2010). This differential equation solver also allowed us to evaluate equilibrium stability. The lsodar function in the package includes a root‐finding method, where we assumed population densities had reached equilibrium once the change in density was less than 10^−5^. To evaluate model equilibriums in realistic scenarios, we consider model parameters that allow coexistence, and that improve stability, to improve model tractability. Furthermore, parameter value scenarios were guided by habitat domain concepts derived from empirical synthesis as described below.

### 2.5 Modeling resource utilization distribution scenarios

We begin with the simplest case (i.e., *full habitat overlap*; Fig. 1d,e), where all prey and predators are each centered at the same point in the habitat (i.e., all θ_*j*_ = μ_*i*_), and the breadth of the predator habitat utilization domains are equal (σ_1_ = σ_2_). In this case, we assume that predators are able to interfere with, but not eat one another. Variation in prey resource utilization allows us to switch from a case where all prey and predators have broadly overlapping resource utilization distribution (Fig. 1d) to a scenario where prey have narrow prey resource utilization distributions nested within that of the predators, in which case empirical synthesis suggests risk enhancement should occur (Fig. 1b). We therefore, evaluate the effects of variation in τ on the equilibrium abundance of each species, and emergent multiple predator effects.

To evaluate the effects of predator habitat partitioning on emergent predator effects, we consider the scenario where predator utilization distributions are narrow and centered at different locations along the habitat gradient (μ_1_ ≠ μ_2_, small σ_*i*_; Fig 1a,c). We assume that predators interfere, but do not consume each other. We consider the case where prey utilization distributions are broad and centered between predator utilization distributions (θ_1_ = θ_2_, large τ; Fig. 1a), or are narrow and separated in space, each aligned with one of the two‐predator utilization distributions (θ_1_ ≠ θ_2_, small τ; Fig. 1c). To evaluate the effects of spatial separation of predators, we evaluate the effects of the distance from the centers of the utilization distributions for predator 1 (μ_1_) and predator 2 (μ_2_) on the equilibrium densities of each species. We consider this variation in predator utilization distributions for the case where prey habitat utilization distributions are always aligned with the corresponding predator utilization distribution (Fig. 1c), and where they remain centered between the two‐predator utilization distributions (Fig. 1a). For the scenario where different prey utilization distributions are each aligned with a different predator utilization distribution (Fig. 1c), we also consider the influence of prey utilization distribution breadth (τ) on equilibrium densities of each species and emergent multiple predator effects. Although the habitat domain describes a single‐prey population (Fig. 1c), for consistency across the scenarios, we evaluate two‐prey populations that have identical distributions. Increases in prey utilization habitat breadth in the scenario where predator habitat distributions differ (μ_1_ ≠ μ_2_) and prey utilization distributions are each centered between the predator distributions (Fig. 1a) always increases predation rates and reduces prey abundance, without impacting multiple predator effects (data not shown), so we do not present this case here. Finally, for the predator habitat partitioning scenario (Fig. 1c), we evaluate the effects of predator habitat breadth (σ_1_ = σ_2_) on the equilibrium abundances of each species, and emergent multiple predator effects.

To evaluate the effects of intraguild predation on emergent multiple predator effects, we consider scenarios where empirical synthesis has most often found intraguild predation to occur (Fig. 1f). In this case, we assume that the predator resource utilization distributions are identical (μ_1_ = μ_2,_ σ_1_ = σ_2_) and that prey population resource utilization distributions are also identical, but not centered at the same location as the predators (θ_1_ = θ_2_ ≠ μ_1_ = μ_2_). We then evaluate the effects of prey resource utilization distribution (τ) on equilibrium abundances of each species and multiple predator effects. In addition, we reconsider the scenario where predators differ in their habitats and prey utilization distributions are each centered on a predator utilization distribution (similar to Fig. 1c), but now include intraguild predation. In this case, we evaluate the effects of the distance between predator habitats, as well as the breadth of predator habitats when separated on equilibrium abundances and multiple predator effects. For the scenario where we alter the distance between predator utilization distributions, we assume that prey populations are held constant at the maximum evaluated predator distance.

## 3 RESULTS

### 3.1 Overlapping utilization distributions

**Figure 3:**
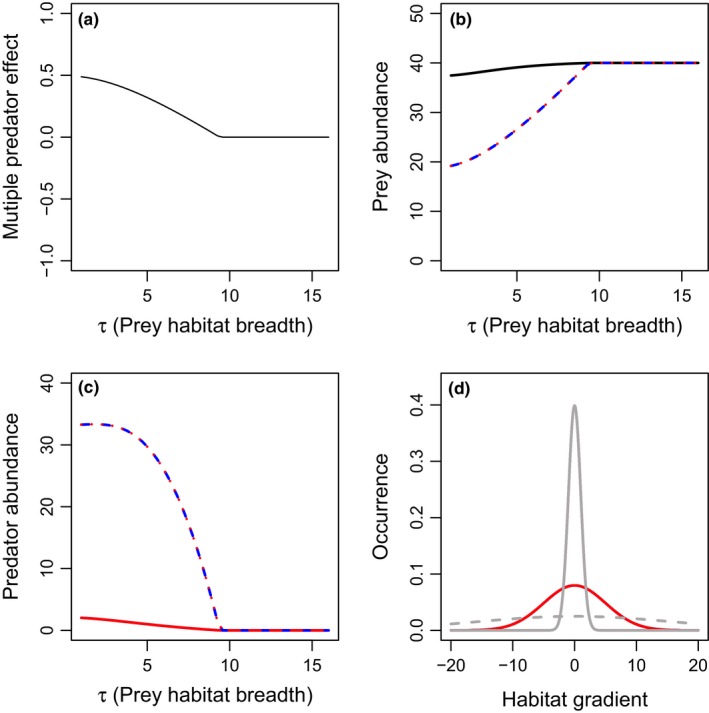
Effects of prey habitat utilization distribution breadth (τ) on multiple predator effects (a) and total densities of prey (b), or predators 1 (blue), and 2 (red) (c), when there are two predators with the same habitat domains (i.e., full habitat overlap). Scenarios include: predator 1 only (blue long‐dashed lines), predator 2 only (red short‐dashed lines), or both predators present (solid lines). The two predators are identical in basal predation and mortality rates. The spatial distribution of the prey (gray) and predators (red) are presented in panel d. Dashed and solid lines in panel d represent the prey distributions for the lowest (solid line) and highest (dashed line) values of τ_*i*_ (τ_1_ = τ_2_) evaluated. Parameter values include *r*_1_ = *r*_2_ = 1, *K*_1_ = *K*_2_ = 20, *c*_1_ = *c*_2_ = 0.2, *m*_1_ = *m*_2_ = 0.06, α_1_ = 0.2, α_2_ = 0.2, β = 0.2, σ_P1_ = σ_P2_ = 5, and θ_1_ = θ_2_ = μ_1_ = μ_2_ = 0. More‐negative multiple predator effects indicate stronger risk‐enhancing effects of multiple predators. Predator densities are identical in the two‐predator scenario, and thus overlap in panel c

As a baseline, we consider the scenario where both predators and the prey species have similar resource utilization distributions (Fig. 1d,e) and evaluate the effect of the prey parameter τ that represents the breadth of a given prey individual's habitat. Reducing the value of τ can move the system from one where predators and prey have broadly overlapping resource utilization distribution (Fig. 1d) to a scenario where prey distributions are narrow and nested within the predators’ (Fig. 1b). When both predator resource utilization distributions perfectly overlap, interference drives strong risk reduction between the two predators (Fig. 3). We find that in this scenario increases in prey habitat utilization distribution breadth, τ reduces predation rates, leading to lower predator, and higher prey densities, eventually leading to predator extinction (Fig. 3). Decreases in τ correspond with decreases in risk reduction, simply due to the effects of decreasing predation rates on prey densities in each scenario (Fig. 3). When predators differ in their attack rates, the better predator drives the other predator to extinction through exploitative competition, and the two‐predator system is equivalent to the single‐predator system (data not shown).

### 3.2 Separate predator distributions

**Figure 4:**
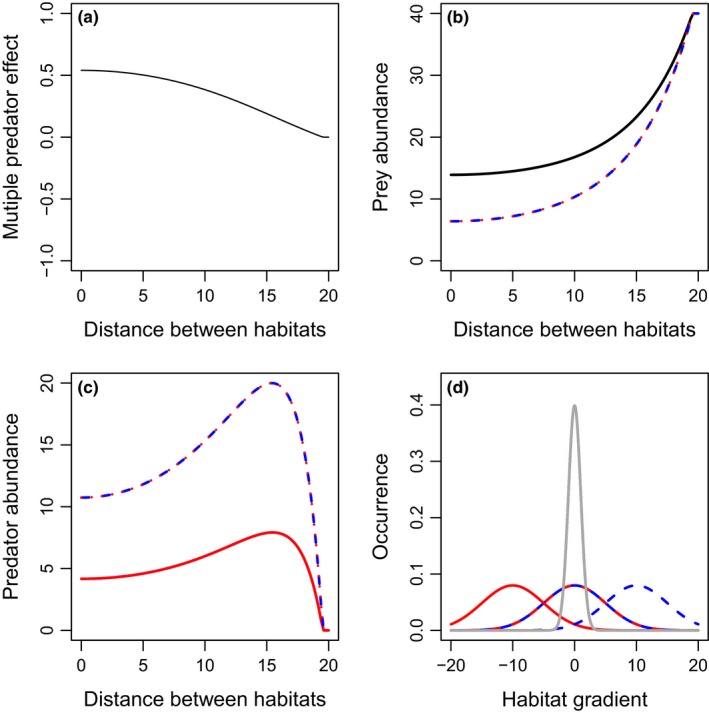
Effects of differences in predator habitat (μ_1_–μ_2_) on multiple predator effects (a) and total densities of prey (b), or predators 1 (blue), and 2 (red) (c), when the two predators have the same habitat domain breadth. In this case, prey habitats shift with predator habitats. Scenarios include: predator 1 only (blue long‐dashed lines), predator 2 only (red short‐dashed lines), or both predators present (solid lines). The two predators are identical in basal predation and mortality rates. The spatial distribution is presented in panel d, with the lower (solid lines) and upper (dashed lines) limits of the habitat positions presented for each prey distribution (gray lines), predator 1 (blue), and predator 2 (red). The two prey species have identical distributions, so only one is shown. Parameter values include *r*_1_ = *r*_2_ = 1, *K*_1_ = *K*_2_ = 20, *c*_1_ = *c*_2_ = 0.2, *m*_1_ = *m*_2_ = 0.1, α_1_ = α_2_ = 1, β = 0.5, θ_1_ = θ_2_ = 0, σ_P1_ = σ_P2_ = 5, and τ_1_ = τ_2_ = 1. More‐negative multiple predator effects indicate stronger risk‐enhancing effects of multiple predators. Predator densities are identical in the two‐predator scenario, and thus overlap in panel c

**Figure 5:**
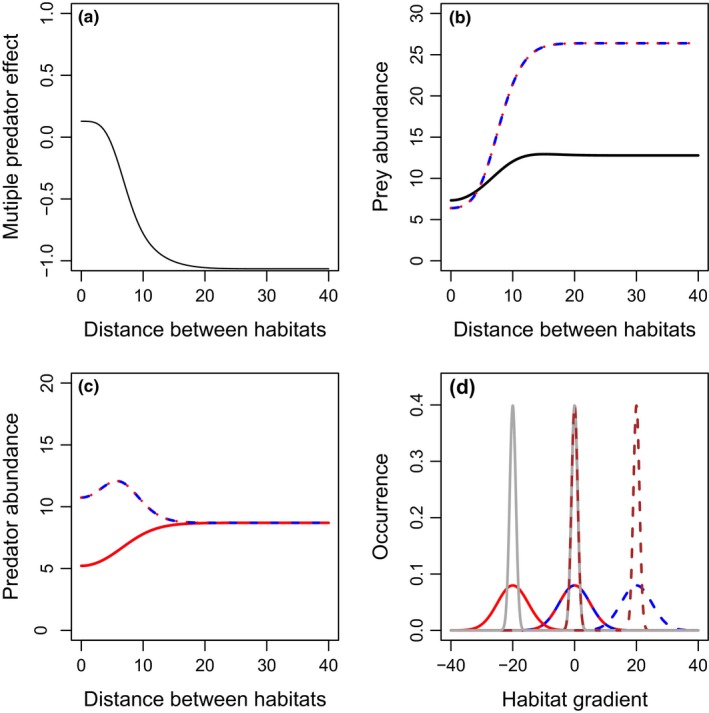
Effects of differences in predator habitat (μ_1_–μ_2_) on multiple predator effects (a) and total densities of prey (b), or predators 1 (blue), and 2 (red) (c), when the two predators have the same habitat domain breadth. In this case, prey habitats remain in center between predators. Scenarios include: predator 1 only (blue long‐dashed lines), predator 2 only (red short‐dashed lines), or both predators present (solid lines). The two predators are identical in basal predation and mortality rates. The spatial distribution is presented in panel d, with the lower (solid lines) and upper (dashed lines) limits of the habitat positions presented for prey 1 (brown), prey 2 (gray), predator 1 (blue), and predator 2 (red). All prey population spatial distributions are identical and thus overlapping in the figure. Parameter values include *r*_1_ = *r*_2_ = 1, *K*_1_ = *K*_2_ = 20, *c*_1_ = *c*_2_ = 0.2, *m*_1_ = *m*_2_ = 0.1, α_1_ = α_2_ = 1, β = 0.05, σ_P1_ = σ_P2_ = 5, and τ_1_ = τ_2_ = 1. More‐negative multiple predator effects indicate stronger risk‐enhancing effects of multiple predators. Predator densities are identical in the two‐predator scenario, and thus overlap in panel c

To evaluate the effects of the distance between predator resource utilization distributions on multiple predator effects, we keep the breadth of the predator (σ_*i*_) and prey (τ) resource utilization distributions constant and vary the distance between predator distributions along the habitat gradient (as in Fig. 1a,c). We consider the case where both prey habitats remain centered at zero (Fig. 1a), and the case where each prey population's distribution shifts with its respective predator (Fig. 1c). In each case, risk reduction declines with increased distance between predator habitats (Figs 4 and 5), due to reduced predator interference. However, it is only in the case where each prey population shifts along with the predator that risk enhancement emerges from separated predator distributions, as shown by negative *D*_*T*_ values (Fig. 5a). This multiple predator effect emerges from steep increases in equilibrium prey abundances in the single‐predator scenarios as the single‐predator habitat is moved far from one of the prey population's resource utilization distribution (Fig 5b). In this case, each predator is necessary for suppression of the prey population with an overlapping distribution. Thus, risk reduction is reduced through reduced interference when predator populations are separated (Figs 4 and 5), but predator habitat partitioning only drives risk enhancement when prey population resource utilization distributions are paired with their respective predators (Fig. 5).

**Figure 6:**
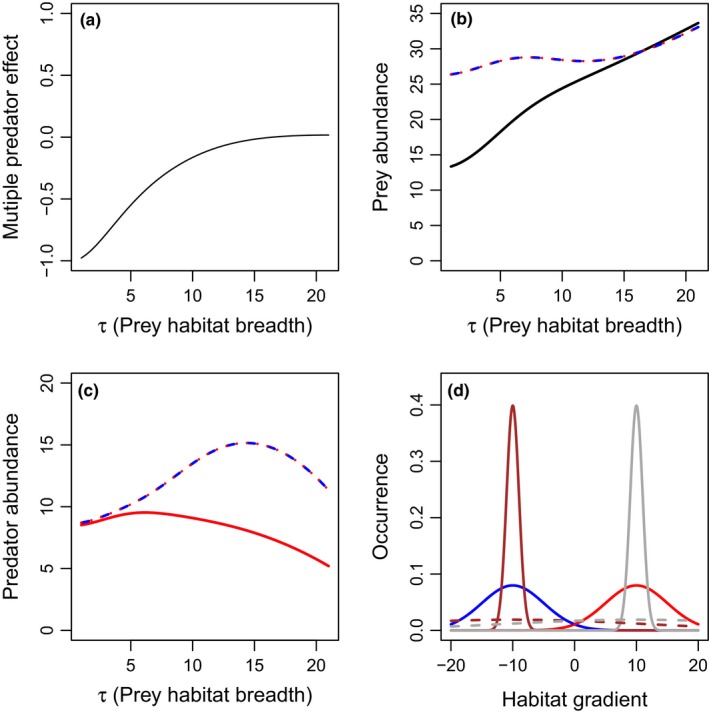
Effects of prey realized habitat spread (τ) on multiple predator effects (a) and total densities of prey (b), or predators 1 (blue), and 2 (red) (c), when there are two predators with the different habitat domains (i.e., habitat partitioning). Scenarios include: predator 1 only (blue long‐dashed lines), predator 2 only (red short‐dashed lines), or both predators present (solid lines). The two predators are identical in basal predation and mortality rates. The spatial distribution of the predators is presented in panel d for predator 1 (blue) and predator 2 (red). Parameter values include *r*_1_ = *r*_2_ = 1, *K*_1_ = *K*_2_ = 20, *c*_1_ = *c*_2_ = 0.2, *m*_1_ = *m*_2_ = 0.1, α_1_ = α_2_ = 1, β = 0.5, and σ_P1_ = σ_P2_ = 5. More‐negative multiple predator effects indicate stronger risk‐enhancing effects of multiple predators. Predator densities are identical in the two‐predator scenario, and thus overlap in panel c

Next, we consider the scenario where predators’ resource utilization distributions spatially separated and prey population centers match the predators (Fig. 1c), and we evaluate the effects of prey habitat utilization breadth, τ, on multiple predator effects. Increases in prey habitat utilization breadth, τ, reduce multiple predator effects (Fig. 6a), because as prey populations become less spatially diffuse the predator in the single‐predator treatment can access both prey populations. This allows the single predator to increase its abundance until the diffusion reduces consumption on the primary prey population (Fig. 6c). Thus, predator niche partitioning does not drive risk enhancement when prey habitat utilization is broad.

**Figure 7:**
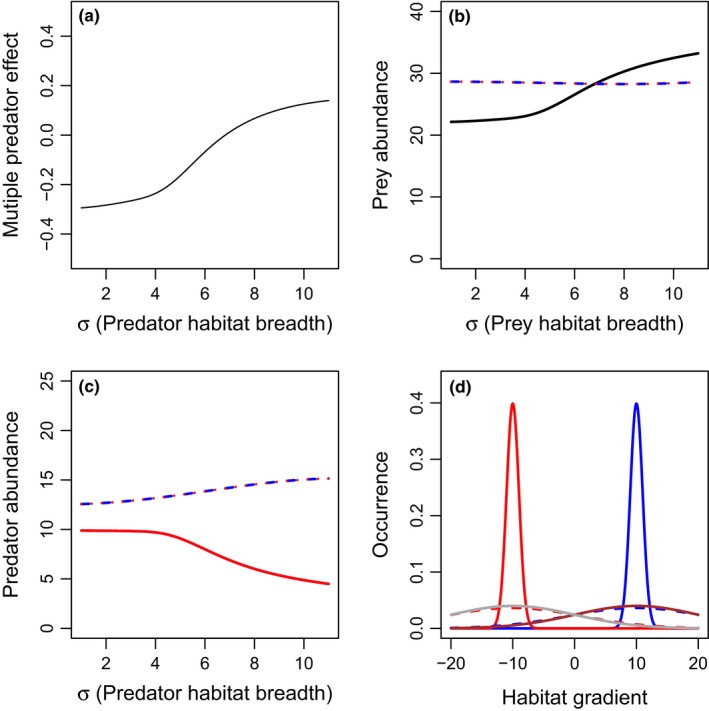
Effects of predator habitat variance (σ_1_ = σ_2_) on multiple predator effects (a) and total densities of prey (b), or predators 1 (blue), and 2 (red) (c), when there are two predators with the different habitat domains (i.e., habitat partitioning). Scenarios include: predator 1 only (blue long‐dashed lines), predator 2 only (red short‐dashed lines), or both predators present (solid lines). The two predators are identical in basal predation and mortality rates. The spatial distribution is presented in panel d, with the lower (solid lines) and upper (dashed lines) limits of the prey habitat breadth presented for prey 1 (brown), prey 2 (gray), predator 1 (blue), and predator 2 (red). All predator population spatial distributions are identical and thus overlapping in the figure. Parameter values include *r*_1_ = *r*_2_ = 1, *K*_1_ = *K*_2_ = 20, *c*_1_ = *c*_2_ = 0.2, *m*_1_ = *m*_2_ = 0.1, α_1_ = α_2_ = 1, β = 0.5, θ_1_ = μ_1_ = 10, θ_2_ = μ_2_ = −10, and τ_1_ = τ_2_ = 10. More‐negative multiple predator effects indicate stronger risk‐enhancing effects of multiple predators. Predator densities are identical in the two‐predator scenario, and thus overlap in panel c

We consider the scenario where prey species have broad but separated resource utilization distributions (τ = 10) and predator species have separated resource utilization distributions (1C) and evaluate the effects of predator resource utilization distribution breadth on multiple predator effects. In the case where predator resource utilization distribution breadths are broad and each prey species is distributed widely across the habitat gradient, predictions should be similar to that described in 1D. Risk enhancement occurs when predator resource utilization distributions are narrow, but increased predator interference reduces predator abundance and drives risk reduction when predator distributions become broad (Fig. 7a). Furthermore, prey abundance in the presence of a single predator species decreases with increasing resource utilization distribution breadth, as the single predator can attack both prey populations (Fig. 7b). Thus, the combination of increased predation in the single‐species treatments and increased predator interference in the two‐predator scenario leads to a switch from risk enhancement to risk reduction with increases in predator resource utilization distribution breadth.

### 3.3 Intraguild predation

**Figure 8:**
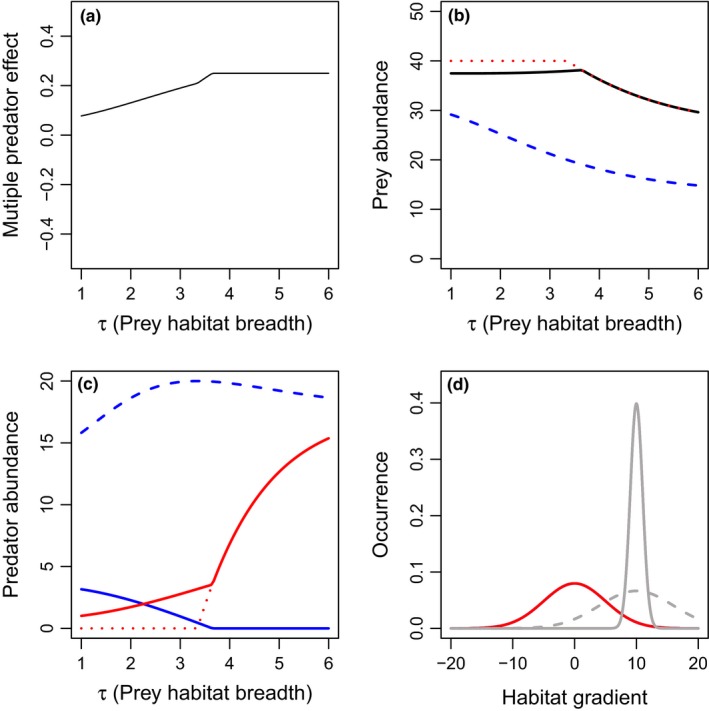
Effects of the parameter τ prey individual habitat breadth, on multiple predator effects (a) and total densities of prey (b), or predators 1 (blue), and 2 (red) (c), when predator two preys on predator 1 and the two predators occupy the same habitat domain nested within the prey habitat. Scenarios include: intraguild prey only (blue long‐dashed lines), intraguild predator only (red short‐dashed lines), or both predators present (solid lines). The spatial distribution is presented in panel d, with the lower (solid lines) and upper (dashed lines) limits of the prey habitat breadth presented for each prey (gray), and predator (red). All predator population spatial distributions are identical and thus overlapping in the figure. Parameter values include *r*_1_ = *r*_2_ = 1, *K*_1_ = *K*_2_ = 20, *c*_1_ = *c*_2_ = 0.2, *m*_1_ = *m*_2_ = 0.1, α_1_ = 1.5, α_2_ = 0.75, β = 0.5, θ_1_ = θ_2_ = 10, μ_1_ = μ_2_ = 0, and σ_1_ = σ_2_ = 5. More‐negative multiple predator effects indicate stronger risk‐enhancing effects of multiple predators

To evaluate the effects of intraguild predation on multiple predator effects, we begin with a scenario that empirical synthesis has generally identified as leading to intraguild predation interactions, where predators have resource utilization distributions nested within prey habitats (Fig. 1f, Schmitz, 2007). We vary the value of τ, the individual prey resource utilization distribution breadth, to move from the scenario described in panel 1e to panel 1f. We assume that the basal predation rate of the intraguild predator is lower than the intraguild prey, which generally improves predator coexistence (Holt & Polis, 1997). Here, we find that as τ increases the predation rate by each predator species increases as well (Fig. 8). However, in the two‐predator scenario, increases in τ shift the balance in predator abundances from intraguild prey‐dominated to intraguild predator‐dominated, eventually driving the intraguild prey extinct (Fig. 8). Because the intraguild prey is the more effective predator, this decrease in intraguild prey density drives risk reduction (Fig. 8a).

**Figure 9:**
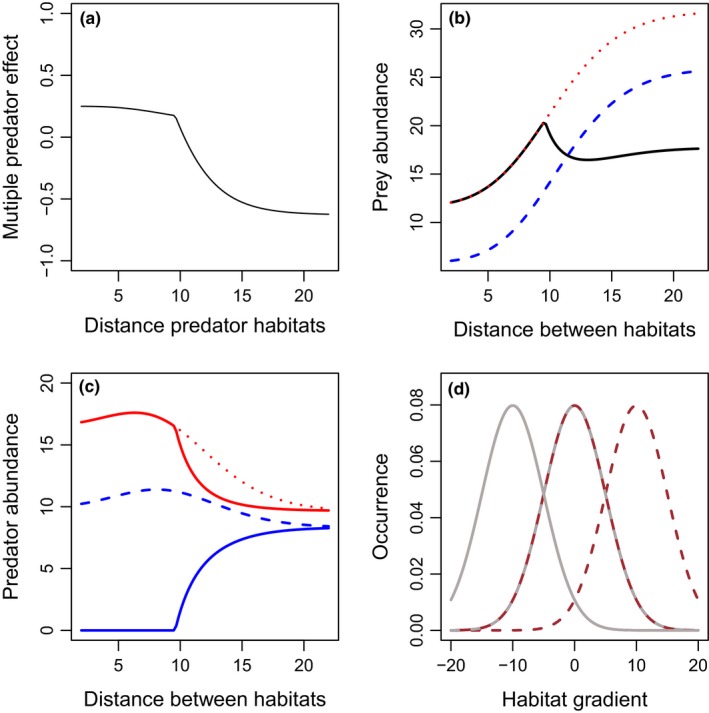
Effects of the distance between predator habitats (μ_1_–μ_2_), on multiple predator effects (a) and total densities of prey (b), or predators 1 (blue), and 2 (red) (c), when predator two preys on predator 1. Scenarios include: intraguild prey only (blue long‐dashed lines), intraguild predator only (red short‐dashed lines), or both predators present (solid lines). The spatial distribution is presented in panel d, with the lower (solid lines) and upper (dashed lines) limits of the distances presented for intraguild prey and prey 1 (brown), and intraguild predator and prey 2 (gray). Predator distributions are identical to that of their associated prey. Parameter values include *r*_1_ = *r*_2_ = 1, *K*_1_ = *K*_2_ = 20, *c*_1_ = *c*_2_ = 0.2, *m*_1_ = *m*_2_ = 0.1, α_1_ = 1.5, α_2_ = 0.75, β = 0.5, θ_1_ = μ_1_, θ_2_ = μ_2_, and τ_1_ = τ_2_ = σ_1_ = σ_2_ = 5. More‐negative multiple predator effects indicate stronger risk‐enhancing effects of multiple predators

**Figure 10:**
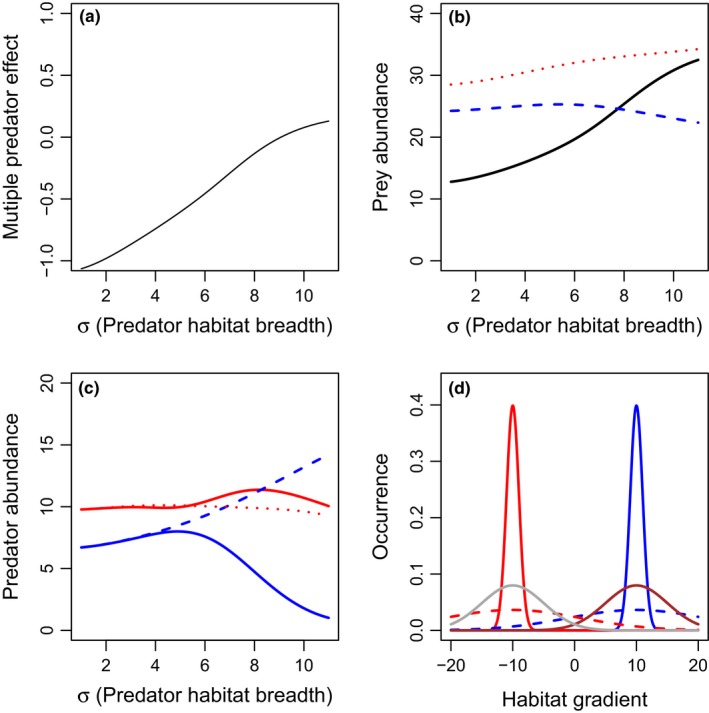
Effects of predator habitat breadth (σ_1_ = σ_2)_, on multiple predator effects (a) and total densities of prey (b), or predators 1 (blue), and 2 (red) (c), when predator two preys on predator 1. Scenarios include: intraguild prey only (blue long‐dashed lines), intraguild predator only (red short‐dashed lines), or both predators present (solid lines). The spatial distribution is presented in panel d, with the lower (solid lines) and upper (dashed lines) limits of the distances presented for intraguild prey (blue) and intraguild predator (red). Prey distributions are presented in brown (prey 1) and gray (prey 2) lines. Parameter values include *r*_1_ = *r*_2_ = 1, *K*_1_ = *K*_2_ = 20, *c*_1_ = *c*_2_ = 0.2, *m*_1_ = *m*_2_ = 0.1, α_1_ = 1.5, α_2_ = 0.75, β = 1, θ_1_ = μ_1_ = 10, θ_2_ = μ_2_ = −10, and τ_1_ = τ_2_ = 5. More‐negative multiple predator effects indicate stronger risk‐enhancing effects of multiple predators

In addition to the effects of prey resource utilization distribution breadth, we evaluated the effects of increased distance between predator habitats, and habitat breadth on emergent multiple predator effects. Although this isn't explicitly described in Fig. 1, evaluating the impact of predator separation on multiple predator effects when intraguild predation can occur will help identify the potential mechanisms, including mechanisms mediated by intraguild predation, driving multiple predator diversity effects in Fig. 1c. In the scenario where prey resource utilization distributions match their associated predators, separating predator (and associated prey) habitats decreases interactions between predators, and the system can shift from risk reduction to risk enhancement (Fig. 9). However, in the scenario with segregated prey and predator populations, increased predator resource utilization distribution breadth can lead to increased predator interactions, higher rates of intraguild predation, and reduced risk enhancement (Fig. 10). Thus, intraguild predation is most likely to drive risk reduction when predators have broadly overlapping habitat domains.

## 4 DISCUSSION

Using a general model, we have shown that emergent multiple predator effects predictably depend on spatial overlap between the resource utilization distribution of the different predators and their prey. Previously Ives, Cardinale, and Snyder (2005) used models to demonstrate that multiple predator effects driving risk enhancement can emerge when predators feed on different species, or when one predator's attack rate increases in the presence of another. In contrast, intraguild predation is expected to drive risk reduction in multiple predator communities when predators share a focal prey species controlled primarily by the intraguild prey (Ives et al., 2005). Here, we build on this theory to demonstrate that the habitat domain concepts built on empirical synthesis generally hold for longer time scales and do not depend on spatial scale. However, we also identify areas where short‐term dynamics that drive experimental studies become less important when the system is allowed to reach equilibrium.

### 4.1 Full habitat overlap and predator antagonism

Our model analyses suggest that increased predator habitat overlap leads to increased predator–predator interactions, which in turn strengthens risk reduction from predator interference or intraguild predation. These results are consistent with the findings of a long history of theoretical studies evaluating predator–prey dynamics in space (Chesson, 2000; Holt & Lawton, 1994; Klopfer & Ives, 1997; Snyder, Borer, & Chesson, 2005; Vanbaalen & Sabelis, 1993). Furthermore, these results support empirical synthesis suggesting that predator antagonism arises when both predator species and prey overlap spatially, and predators and prey all have large habitat domain (Fig. 1d), or when they all have small habitat domains (Fig. 1e) (Schmitz, 2007). In each case, empirical synthesis suggests predators undergo interference competition, because one predator species preempts the other from gaining access to the shared prey. The predators then reduce their net effects on the prey by engaging in interference interactions (Schmitz, 2007). In our model analyses, we assume that interference‐driven mortality is a function of spatial overlap, such that predators that interact spatially are more likely to interfere with one another (as in May, 2001). Therefore, the mechanisms driving model predictions depend on the assumption that predators are more likely to interfere when scramble competition for prey is greater, due to greater spatial overlap in resource utilization distributions, which aligns with findings from empirical systems. Although the habitat domain concepts describe interference driving these mechanisms, our model analyses suggest that the general findings do not depend on the type of antagonism between predators (i.e., intraguild predation versus interference).

When both predator resource utilization distributions perfectly overlap, we find reducing prey habitat utilization distribution breadth (τ) leads to lower predation rates and higher prey abundance, but has no effect on emergent multiple predator effects (Fig. 3a). This is in contrast to the results generally found in short‐term experiments, where predators often combine to increase mortality risk to prey, relative to their individual effects (Schmitz, 2007). In empirical systems, this risk enhancement by multiple predators arises because predators can forage widely and supplement their diet with alternative prey that they are unique to each predator (Schmitz, 2007). Thus, the risk enhancement for the shared, focal prey is driven by numerical responses from alternative prey outside the shared habitat. We do not explicitly consider alternative prey here, and therefore, this mechanism cannot occur.

### 4.2 Predator habitat partitioning

We find that predator niche partitioning stemming from increased distance between resource utilization distributions and reduced distribution breadth can increase prey consumption in diverse predator habitats in two ways. First, increasing the habitat segregation can reduce predator–predator interactions, thus reducing risk reduction through either interference or intraguild predation. When prey habitat domains span across the two‐predator habitats (Fig. 1a) this predator habitat partitioning can lead to substitutive effects of the different predators (Figs 5, 9 and 10). Empirical synthesis suggests the same mechanisms reduce risk reduction when predators inhabit different habitat domains, but prey habitat domain is large (Fig. 1a) (Schmitz, 2007). In this case, predators have substitutive effects on prey because, by being in separate locations, one predator spatially compensates for the effects of the other predator (Schmitz, 2007). However, spatial separation reduces any direct negative interactions between predators that might reduce predation rates. In contrast, increasing the distance between predator habitats can lead to risk enhancement when there are two‐prey populations that are each constrained to one of the two‐predator habitats (Figs 1c and 5). Similarly, empirical synthesis suggests that risk enhancement occurs when predators inhabit different habitat domains and prey occupy distinct, narrow domains (Northfield, Crowder et al., 2012). In this case, the two predators technically do not compete, but rather operate as separate food chains involving their spatially corresponding prey population, leading to enhanced risk of mortality to the entire prey species across the landscape (Northfield, Crowder et al., 2012). In our models, this segregation of prey habitats occurs when the parameter τ is reduced, which biologically can be driven by reduced prey movement along the habitat gradient (Fig. 6).

### 4.3 Prey refuge habitat

Synthesis of empirical literature suggests that intraguild predation drives risk reduction when prey have a broad habitat resource utilization distributions and predators have narrow, overlapping habitat domain, and different hunting modes (Schmitz, 2007). In this case, prey can avoid predators by persisting in the predator‐free refuge, and without their preferred prey, the intraguild predator then attacks the intraguild prey. Here, we consider the scenario where prey have a habitat domain that extends beyond that of the predator (Fig. 1f) and evaluate the effect of increasing prey habitat breadth on multiple predator effects. We find that in the two‐predator scenario when intraguild predation occurs, increasing prey habitat breadth from very low values increased the predation rate of the intraguild prey, which then leads to increased rates of intraguild predation, and increased densities of the intraguild predator. This intraguild predation drives increases in risk reduction with increases in prey habitat breadth (Fig. 8). If the prey habitat breadth is large enough, predation on prey alone can support the top predator, and it drives the intraguild prey extinct. The inclusion of a broad prey habitat domain that extends beyond the predator domain increases intraguild predation, and in turn, risk reduction through numerical effects that travel up the food chain. While the results from our modeling analysis matches the results generally found in experiments (e.g., Barton & Schmitz, 2009), the mechanisms are quite different. Here, we find that the results are mediated by dynamical processes that may occur over timelines longer than those typical of mesocosm experiments, while the results in mesocosm experiments are driven by behavioral interactions not considered in our models. Therefore, while the theoretical and empirical results are similar, it is important to note the difference in mechanisms when applying them to empirical systems.

### 4.4 Considerations for future research

One strength of our modeling framework is the ease with which additional biological realisms can be added. For example, here, we did not consider nonconsumptive effects where predators induce a change in prey resource utilization distribution, which is often found in predator–prey systems (e.g., Schmitz, 2008). However, these trait‐mediated interactions could easily be added to the model by making the center (θ) and/or breadth (τ) of the prey resource utilization distribution dependent on one or both predator species, or by allowing prey to adapt their habitat domain according to rules such as quantitative genetics models that could describe evolutionary or plastic change (see Abrams, 2000 for review). Furthermore, allowing rapid trait changes in the predators may lead to alternative stable states, or stabilize unstable ecological dynamics (Patel & Schreiber, 2015). Other environmental axes could also be included in models with either stationary or dynamic habitat domains by modeling habitat domains as multivariate Gaussian distributions. Application of recent theoretical research on eco‐evolutionary dynamics of a consumer–resource system governed by generic species traits suggests that when resource utilization distributions are dynamic, an increase in the number of axes describing habitat gradients would generally increase prey survival, essentially by increasing potential for enemy‐free space (Gilman, Nuismer, & Jhwueng, 2012).

Our modeling framework could also be altered to include additional prey or predators in the food web. For example, risk enhancement between predators often occurs when predators have wide habitat domains, but prey has narrow habitat domains (Schmitz, 2007). This can occur, because predators each have alternative prey over which they do not compete, allowing them to reach high abundances and maintain low prey densities. Although considering this scenario would involve the inclusion of other prey species, the modeling framework could easily be altered to do so.

## 5 CONCLUSION

We used a theoretical model describing the resource utilization distributions of predators and their shared prey to generate theory describing when multiple predators combine to enhance versus reduce predation risk. The model is based on early concepts of niches as Gaussian distributions over a habitat gradient (e.g., May, 2001) and has been repurposed to evaluate multiple predator effects. These model predictions align with empirical results from mesocosm experiments. In addition, the analytical results present a theoretical framework capable of demonstrating multiple predator effects that does not depend on the small spatial or temporal scales typical of mesocosm experiments, and can help bridge between empirical experiments and long‐term dynamics in natural systems (Schmitz, 2007).

## CONFLICT OF INTEREST

None declared.

## AUTHOR CONTRIBUTIONS

All authors contributed to the conceptualization. Model analyses were conducted by TDN with input from OJS and BTB. The manuscript was principally written by TDN and OJS input from BTB.
