# Supplementary material for: A spatial theory for emergent multiple predator–prey interactions in food webs
Source: Ecol Evol. 2017 Jul 28;7(17):6935–48. doi: 10.1002/ece3.3250 (PMC5587500; doi:10.1002/ece3.3250)
Supplement: Supplementary file 1 [file ECE3-7-6935-s001.docx]

Appendix S1. Representative equilibrium solutions

We use the term *b_i_* to define the effect of predator interference on predator species *i*. By tracking the terms separately, each equilibrium solution can easily be altered for the intraguild predation model. We present equilibrium solutions for the two prey species (N_1_^*^ and N_2_^*^), and two predator species (P_1_^*^ and P_2_^*^), when they are non-zero. Equations for capital letters on the right hand side are presented below each equilibrium solution.

2 prey, 2 predator equilibrium.

Prey 1:

$$N_{1}^{*}=\frac{k_{1}r_{2}A_{1}+k_{1}k_{2}\left[ \bar{a}_{12}\bar{a}_{22}B_{1}-\bar{a}_{12}\bar{a}_{21}c_{1}C_{1}-\bar{a}_{11}\bar{a}_{22}c_{2}D_{1} \right]}{b_{1}b_{2}r_{1}r_{2}+k_{1}r_{2}E+k_{2}r_{1}F-k_{1}k_{2}c_{1}c_{2}G}$$

*A*_1_ = *ā*_11_*b*_1_*m*_2_ + *ā*_21_*b*_2_m_1_+ *b*_1_ *b*_2_r_1_

*B*_1_ = *b*_1_*c*_2_*r*_1_ + *b*_2_*c*_1_*r*_1_ + *ā*_11_*c*_1_*m*_2_ + *ā*_21_*c*_2_*m*_1_

*C*_1_ = *ā*_12_*m*_2_+*b*_2_*r*_2_

*D*_1_ = *ā*_22_*m*_1_+ *b*_1_*r*_2_

*E* = *ā*_11_*ā*_21_(*b*_1_*c*_2_+*b*_2_*c*_1_)

*F* = *ā*_12_*ā*_22_(*b*_1_*c*_2_+*b*_2_*c*_1_)

*G* = (*ā*_11_*a*_22_ - *ā*_12_*ā*_21_)^2^

Prey 2:

$$N_{2}^{*}=\frac{k_{2}r_{1}A_{2}+k_{1}k_{2}\left[ \bar{a}_{21}\bar{a}_{11}B_{2}-\bar{a}_{21}\bar{a}_{12}c_{2}C_{2}-\bar{a}_{11}\bar{a}_{22}c_{1}D_{2} \right]}{b_{1}b_{2}r_{1}r_{2}+k_{1}r_{2}E+k_{2}r_{1}F-k_{1}k_{2}c_{1}c_{2}G}$$

*A_2_* = *ā* _22_*b*_2_*m*_1_ + *ā*_12_*b*_1_m_2_+ *b*_1_ *b*_2_r_2_

*B_2_* = *b*_2_*c*_1_*r*_2_ + *b*_1_*c*_2_*r*_2_ + *ā*_22_*c*_2_*m*_1_ + *ā*_12_*c*_1_*m*_2_

*C_2_* = *ā*_21_*m*_1_+*b*_1_*r*_1_

*D_2_* = *ā*_11_*m*_2_+ *b*_2_*r*_1_

*E* = *ā*_11_*ā*_21_(*b*_1_*c*_2_+*b*_2_*c*_1_)

*F* = *ā*_12_*ā*_22_(*b*_1_*c*_2_+*b*_2_*c*_1_)

*G* = (*ā*_11_*a*_22_ - *ā*_12_*ā*_21_)^2^

Predator 1:

$$P_{1}^{*}=\frac{k_{1}r_{2}a_{21}H_{1}+k_{2}r_{1}a_{22}I_{1}+k_{1}k_{2}c_{1}c_{2}J_{1}{-b}_{1}m_{2}r_{1}r_{2}}{b_{1}b_{2}r_{1}r_{2}+k_{1}r_{2}E+k_{2}r_{1}F-k_{1}k_{2}c_{1}c_{2}G}$$

*H_1_* = *ā*_21_*c*_2_*m*_1_ - *ā*_11_*c*_1_m_2_ + *b*_1_*c*_2_r_1_

*I_1_* = *ā*_22_*c*_2_*m*_1_ - *ā*_12_*c*_1_m_2_ + *b*_1_*c*_2_r_2_

*J_1_* = *ā_11_ā_21_ā_22_r_2_ + ā_12_ā_21_ā_22_r_1_* - *ā_11_ā_22_^2^r_1_* - *ā_12_ā_21_^2^r_2_*

*E* = *ā*_11_*ā*_21_(*b*_1_*c*_2_+*b*_2_*c*_1_)

*F* = *ā*_12_*ā*_22_(*b*_1_*c*_2_+*b*_2_*c*_1_)

*G* = (*ā*_11_*ā*_22_ - *ā*_12_*ā*_21_)^2^

Predator 2:

$$P_{2}^{*}=\frac{k_{2}r_{1}a_{12}H_{2}+k_{1}r_{2}a_{11}I_{2}+k_{1}k_{2}c_{1}c_{2}J_{2}{-b}_{2}m_{1}r_{1}r_{2}}{b_{1}b_{2}r_{1}r_{2}+k_{1}r_{2}E+k_{2}r_{1}F-k_{1}k_{2}c_{1}c_{2}G}$$

*H_2_* = *ā*_12_*c*_1_*m*_1_ - *ā*_22_*c*_2_m_1_ + *b*_2_*c*_1_r_2_

*I_2_* = *ā*_11_*c*_1_*m*_2_ - *ā*_21_*c*_2_m_1_ + *b*_2_*c*_1_r_1_

*J_2_* = *ā_11_ā_12_ā_22_r_1_ + ā_11_ā_12_ā_21_r_2_* - *ā_11_^2^a_22_r_2_ - ā_12_^2^ā_21_r_1_*

*E* = *ā*_11_*ā*_21_(*b*_1_*c*_2_+*b*_2_*c*_1_)

*F* = *ā*_12_*ā*_22_(*b*_1_*c*_2_+*b*_2_*c*_1_)

*G* = (*ā*_11_*ā*_22_ - *ā*_12_*ā*_21_)^2^

1 prey, 2 predators equilibrium:

$$N_{1}^{*}=\frac{k_{1}\left( \bar{a}_{11}b_{1}m_{2}+\bar{a}_{21}b_{2}m_{1}+b_{1}b_{2}r_{1} \right)}{b_{1}b_{2}r_{1}+k_{1}\bar{a}_{11}\bar{a}_{21}\left( b_{1}c_{2}+b_{2}c_{1} \right)}$$

$$P_{1}^{*}=\frac{k_{1}\bar{a}_{21}\left( \bar{a}_{21}c_{2}m_{1}-\bar{a}_{11}c_{1}m_{2}+b_{1}c_{2}r_{1} \right)-b_{1}m_{2}r_{1}}{b_{1}b_{2}r_{1}+k_{1}\bar{a}_{11}\bar{a}_{21}\left( b_{1}c_{2}+b_{2}c_{1} \right)}$$

$$P_{2}^{*}=\frac{k_{1}\bar{a}_{11}\left( \bar{a}_{11}c_{1}m_{2}-\bar{a}_{21}c_{2}m_{1}+b_{2}c_{1}r_{1} \right)-b_{2}m_{1}r_{1}}{b_{1}b_{2}r_{1}+k_{1}\bar{a}_{11}\bar{a}_{21}\left( b_{1}c_{2}+b_{2}c_{1} \right)}$$

2 prey, 1 predator equilibrium:

$$N_{1}^{*}=\frac{k_{1}\left( \bar{a}_{11}r_{2}m_{1}+k_{2}\bar{a}_{12}\left( \bar{a}_{12}c_{1}r_{1}-\bar{a}_{11}c_{1}r_{2} \right) \right)}{c_{1}\left( k_{1}{\bar{a}_{11}}^{2}r_{2}+k_{2}{\bar{a}_{12}}^{2}r_{1} \right)}$$

$$N_{2}^{*}=\frac{k_{2}\left( \bar{a}_{12}r_{1}m_{1}+k_{1}\bar{a}_{11}\left( \bar{a}_{11}c_{1}r_{2}-\bar{a}_{12}c_{1}r_{1} \right) \right)}{c_{1}\left( k_{1}{\bar{a}_{11}}^{2}r_{2}+{k_{2}\bar{a}_{12}}^{2}r_{1} \right)}$$

$$P_{1}^{*}=\frac{k_{1}\bar{a}_{11}c_{1}r_{1}r_{2}+k_{2}\bar{a}_{12}c_{1}r_{1}r_{2}-m_{1}r_{1}r_{2}}{k_{1}{\bar{a}_{11}}^{2}c_{1}r_{2}+k_{2}{\bar{a}_{12}}^{2}c_{1}r_{1}}$$
